# Supplementary material for: Bayesian refinement of protein structures and ensembles against SAXS data using molecular dynamics
Source: PLoS Comput Biol. 2017 Oct 18;13(10):e1005800. doi: 10.1371/journal.pcbi.1005800 (PMC5662244; doi:10.1371/journal.pcbi.1005800)
Supplement: S1 Archive — (BZ2) [file pcbi.1005800.s011.tar.bz2 › gromacs_4.62_bayesian_saxs_refi/share/html/online/g_dos.html]

g\_dos


| g\_dos Main Table of Contents |

**VERSION 4.6  
Sat 19 Jan 2013**

---

### Description

g\_dos computes the Density of States from a simulations.
In order for this to be meaningful the velocities must be saved
in the trajecotry with sufficiently high frequency such as to cover
all vibrations. For flexible systems that would be around a few fs
between saving. Properties based on the DoS are printed on the
standard output.

### Files

| option | filename | type | description |
| --- | --- | --- | --- |
| **-f** | traj.trr | Input | Full precision trajectory: trr trj cpt |
| **-s** | topol.tpr | Input | Run input file: tpr tpb tpa |
| **-n** | index.ndx | Input, Opt. | Index file |
| **-vacf** | vacf.xvg | Output | xvgr/xmgr file |
| **-mvacf** | mvacf.xvg | Output | xvgr/xmgr file |
| **-dos** | dos.xvg | Output | xvgr/xmgr file |
| **-g** | dos.log | Output | Log file |

### Other options

| option | type | default | description |
| --- | --- | --- | --- |
| **-[no]h** | bool | no | Print help info and quit || **-[no]version** | bool | no | Print version info and quit || **-nice** | int | 19 | Set the nicelevel || **-b** | time | 0 | First frame (ps) to read from trajectory || **-e** | time | 0 | Last frame (ps) to read from trajectory || **-dt** | time | 0 | Only use frame when t MOD dt = first time (ps) || **-[no]w** | bool | no | View output .xvg, .xpm, .eps and .pdb files || **-xvg** | enum | xmgrace | xvg plot formatting: xmgrace, xmgr or none || **-[no]v** | bool | yes | Be loud and noisy. || **-[no]recip** | bool | no | Use cm^-1 on X-axis instead of 1/ps for DoS plots. || **-[no]abs** | bool | no | Use the absolute value of the Fourier transform of the VACF as the Density of States. Default is to use the real component only || **-[no]normdos** | bool | no | Normalize the DoS such that it adds up to 3N. This is a hack that should not be necessary. || **-T** | real | 298.15 | Temperature in the simulation || **-acflen** | int | -1 | Length of the ACF, default is half the number of frames || **-[no]normalize** | bool | yes | Normalize ACF || **-P** | enum | 0 | Order of Legendre polynomial for ACF (0 indicates none): 0, 1, 2 or 3 || **-fitfn** | enum | none | Fit function: none, exp, aexp, exp\_exp, vac, exp5, exp7, exp9 or erffit || **-ncskip** | int | 0 | Skip this many points in the output file of correlation functions || **-beginfit** | real | 0 | Time where to begin the exponential fit of the correlation function || **-endfit** | real | -1 | Time where to end the exponential fit of the correlation function, -1 is until the end |

### Known problems

- This program needs a lot of memory: total usage equals the number of atoms times 3 times number of frames times 4 (or 8 when run in double precision).

---

http://www.gromacs.org  
gromacs@gromacs.org
